# Identification and Characterization of the Vitellogenin Receptor Gene and Its Role in Reproduction in the Alligatorweed Flea Beetle, *Agasicles hygrophila*

**DOI:** 10.3389/fphys.2019.00969

**Published:** 2019-07-31

**Authors:** Hong Zhang, Yiran Liu, Jisu Jin, Zhongshi Zhou, Jianying Guo

**Affiliations:** State Key Laboratory for Biology of Plant Diseases and Insect Pests, Institute of Plant Protection, Chinese Academy of Agricultural Sciences, Beijing, China

**Keywords:** *Agasicles hygrophila*, vitellogenin receptor, RNAi, ovarian development, fecundity

## Abstract

*Agasicles hygrophila* is an effective biological control agent for the invasive weed *Alternanthera philoxeroides*, and because of this it has been introduced to many parts of the world where *A. philoxeroides* is a problem. Despite this, there are no reports at present about the reproduction of this important insect. Vitellogenin receptors (VgRs) belong to the superfamily of low-density lipoprotein receptors (LDLRs). One of the roles of VgRs is to regulate the absorption of yolk protein in insects. In this study, the full length vitellogenin receptor gene (*AhVgR*) from *A. hygrophila* was sequenced and found to encode a predicted protein of 1,642 amino acids. Sequence analysis of AhVgR revealed that it contains conserved structural motifs common to LDLR family members, and a phylogenetic analysis placed AhVgR as a separate group among the order Coleoptera. *AhVgR* was found to be specifically expressed in ovarian tissues, and it is first transcribed in the newly-emerged females. The expression patterns are consistent with *VgR* genes in other insects. RNA interference (RNAi)-mediated suppression of *AhVgR* gene expression in adult *A*. *hygrophila* females inhibited yolk protein deposition in the ovaries, shortened the ovariole, drastically reduced egg production, and ultimately led to a decrease in fecundity. In summary, our work shows that AhVgR is critical for transporting Vg into the oocytes and plays an important role in *A. hygrophila* reproduction.

## Introduction

*Alternanthera philoxeroides*, (Mart.) Griseb (Amaranthaceae), commonly known as alligatorweed, is a perennial aquatic plant native to South America that has invaded many regions of the world, including North America, Asia, and Australia (Julien et al., 1995; Xu et al., 2003). In China, *A. philoxeroides* was introduced as a forage crop in the late 1930s, and subsequently spread to the country’s eastern and southern regions (Ye et al., 2010). *A*. *philoxeroides* is highly competitive, and causes economic and ecological problems almost everywhere it is found (Maddox and Rhyne, 1975). *Agasicles hygrophila* (Selman & Vogt) (Coleoptera: Chrysomelidae), the alligatorweed flea beetle, has proven to be an efficient control agent for *A. philoxeroides* (Buckingham, 1996). The beetle was introduced into China from Florida in 1987, and was first released in Chongqing, Fuzhou, and Changsha in 1988. *Agasicles hygrophila* became established in the release sites and is now distributed mostly in southern China, where it has controlled *A. philoxeroides* for over 30 years (Guo et al., 2011).

As the precursor of the major yolk protein vitellin (Vn), vitellogenin (Vg) provides nutrition necessary for egg development, and plays an important role in insect reproduction (Tufail and Takeda, 2009). During reproduction, Vg is primarily synthesized in the fatbody and then secreted into the hemolymph, where it is absorbed by the developing oocytes via the vitellogenin receptor (VgR) (Lu et al., 2015). Thus, VgR plays an essential role in the process of oocyte maturation (Sappington and Raikhel, 1998). In insects, the *VgR* gene encodes a large ovary-specific protein of 180–214 kDa, which is twice the size of the vertebrate VgR proteins (Sappington and Raikhel, 1998). The amino acid sequences of insect VgRs show that they are members of the low-density lipoprotein receptor (LDLR) family, with two ligand binding domains (LBDs) containing several LDLR-domain class A repeats. The function of VgR is different from than that of Vg, but silencing either of the genes can lead to malformed ovaries and decreased fecundity, as has been shown in *Helicoverpa armigera* (Zhang et al., 2016), *Nilaparvata lugens* (Lu et al., 2015), *Spodoptera litura* (Shu et al., 2011), and *Aphis citricidus* (Shang et al., 2018).

Although VgRs have been described in other insects, there are no studies that describe the VgR of *A. hygrophila*. In our study, we characterized the AhVgR gene and explore its expression patterns in *A. hygrophila*. We cloned the complete sequence of *AhVgR*, and showed its molecular characteristics, phylogenetic relationships and expression patterns. Furthermore, we verified the function of *AhVgR* in ovary development and reproduction through RNA interference experiments.

## Materials and Methods

### Insect Rearing and Sample Collection

*Alternanthera philoxeroides* plants were collected at the Institute of Plant Protection, Hunan Academy of Agricultural Sciences, and grown in the greenhouse at Langfang Experimental Station, Chinese Academy of Agricultural Sciences. Plants were grown in sterilized soil in plastic boxes (40 cm × 18 cm × 15 cm) and watered every other day. *Agasicles hygrophila* adults were initially collected from the field in Changsha. The population was reared on *A. philoxeroides* plants in the laboratory at the Chinese Academy of Agricultural Sciences (Beijing, China) under controlled conditions (28 ± 1°C, 12 h light:dark photoperiod, relative humidity [RH] = 75 ± 5%) (Guo et al., 2011).

To clone the *AhVgR* gene sequence, the ovaries of *A. hygrophila* females were dissected in 1X PBS (phosphate-buffered saline, pH 7.4) using an Olympus stereomicroscope (SZX16, Olympus, Tokyo, Japan). For tissue-specific expression of *AhVgR*, the head, thorax, ovary, fat body, midgut, and wing tissues of 6-day-old beetles were also dissected in PBS using the Olympus SZX16 stereomicroscope. To analyze the expression of the different developmental stages, freshly pupated pupa were collected daily, and female adults were collected every 2 day after emergence. All samples were immediately frozen in liquid nitrogen and stored at −80°C until they were used for RNA isolation.

### RNA Isolation and Gene Cloning

Total RNA was extracted from *A. hygrophila* tissues using TRIzol reagent (Life Technologies, Carlsbad, CA, United States) according to the manufacturer’s protocol. The concentrations of the RNA samples were determined with a NanoDrop ND-1000 spectrophotometer (Thermo Scientific, Wilmington, DE, United States), while degradation and genomic DNA contamination were monitored by electrophoresis through 1% agarose gels. First-strand cDNA from the different RNA samples was synthesized using the TransScript One-Step gDNA Removal and cDNA Synthesis SuperMix Kit (TransGen, Beijing, China) following the manufacturer’s protocol. Our lab mined the *A. hygrophila* transcriptome data to obtain expressed sequence tags (ESTs) that showed similarities to other insect vitellogenin receptor genes. The PCR primers were designed based on the *A. hygrophila* vitellogenin receptor gene cDNA fragment from the transcriptome (Supplementary Table 1). Positive clones were confirmed by PCR amplification and then sequenced. To obtain full-length cDNA sequence of the *AhVgR* gene, we used a SMART^TM^ RACE cDNA amplification kit (Clontech, Mountain View, CA, United States) to amplify the 5′- and 3′-ends. Gene-specific primers were designed (Supplementary Table 1), and the RACE cycling conditions were performed as described in Zhang et al. (2016). The RACE products were then gel-purified and sequenced.

### Gene Sequence Analysis and Phylogenetic Relationships

Sequence similarities were analyzed using the BLAST website^1^. The open reading frames (ORFs) of the *AhVgR* clones were predicted using the NCBI ORF finder^2^. The molecular weights and isoelectric points (pIs) of the inferred AhVgR protein sequences were predicted with the ExPASy proteomics server^3^. The conserved domains and signal peptide were analyzed by the online SMART server^4^ and the SignalP 4.1 Server^5^. The VgR amino acid sequences from other insects were downloaded from the GenBank database and used to in the phylogenetic analyses. The VgR sequence alignments were carried out using Clustal X software. A phylogenetic tree was constructed with molecular evolutionary genetics analysis software (MEGA version 5.0), using the neighbour-joining (NJ) method with a bootstrap test of 1,000 replicates (Tamura et al., 2011).

#### Synthesis of Double-Stranded RNA (dsRNA) and Injection of dsRNA

dsRNA was synthesized from the *AhVgR* cDNA, and *EGFP* (Enhanced Green Fluorescent Protein, GenBank Accession No. AIR08541.1) dsRNA was synthesized as a negative control. To ensure the interference effect, we synthesized two dsRNA fragments from *AhVgR* (ds*VgR*-A and ds*VgR*-B) and *EGFP* (ds *EGFP*- and, ds *EGFP*-B). The target gene dsRNAs were synthesized and purified using the HiScribe^TM^ T7 Quick High Yield RNA Synthesis Kit (New England BioLabs, Ipswich, MA, United States #E2050S) as directed by the manufacturer. The purity of the dsRNA was checked by electrophoresis on a 1.0% agarose gel and the concentration of the dsRNA was measured on a NanoDrop ND-2000 Spectrophotometer (Nanodrop Technologies, Wilmington, DE, United States). To ensure the injection volume between the control group and treatment group was consistent, we adjusted the concentration of all synthesized dsRNA to 10,000 ng/μl. Newly-emerged adult females were collected, and the dsRNA was injected into the conjunctivum using a PLI-100 Pico-Injector (Harvard Apparatus, Holliston, MA, United States) with an MP-255 Micromanipulator (Sutter, Novato, CA, United States) under an Olympus stereomicroscope. Each experiment was repeated three times. Each repeat consisted of 60 female individuals for later experimental observation after injection. The injection dose was 0.1 μl of each dsRNA (ds*VgR* group: ds*VgR*-A 0.1 μl, ds*VgR*-B 0.1 μl; ds*EGFP* group: ds*EGFP*-A 0.1 μl, ds*EGFP*-B 0.1 μl). After injection, female adults were kept in separate plastic bottles (8 cm × 10 cm) and paired with freshly emerged wild-male adults. Each bottle was kept moist with a piece of moistened filter paper at the bottom, and fresh *A. philoxeroides* stems were added.

#### qPCR Experiments

Total RNA was extracted from the samples with TRIzol reagent (Life Technologies, Carlsbad, CA, United States) following the manufacturer’s instructions. First-strand cDNA was synthesized by following the procedures mentioned above. Quantitative real-time PCR (qPCR) analysis was performed with the TransStart Green qPCR SuperMix Kit (Transgen, Beijing, China) on an ABI Prism 7500 system (Applied Biosystems, Carlsbad, CA, United States). *CoxI* (GenBank Accession No. FJ977926.1) was used as an internal control gene. Specific primers were designed for qPCR with Beacon Designer 7.9 software (PREMIER Biosoft International, Davis, CA, United States) (Supplementary Table 1). Thermal cycler amplification conditions were as recommended by the manufacturer. The qPCR reactions were performed in 20 μl volumes containing 10 μl of 2X TransStart Green qPCR SuperMix, 0.4 μl of each primer (10 μM), 0.4 μl of 50X Rox Reference Dye, 200 ng of sample cDNA, and 7.8 μl of ddH_2_O. The qPCR cycling parameters were as follows: 94°C for 30 s, followed by 40 cycles of 94°C for 30 s, 55°C for 30 s, and 72°C for 10 s, with melt curve stages at 95°C for 15 s, 60°C for 1 min, and 9°C for 15 s. To ensure reliability, each reaction for each sample was performed three times with three technical replicates. Each run included negative controls with no cDNA template. The relative expression of each gene was calculated using the 2^–ΔΔCT^ method of Livak and Schmittgen (2001).

#### Observation of Ovary Development and Fecundity After RNAi

Each dsRNA-treated *A. hygrophila* female was observed 4 days after dsRNA injection to examine ovary development. The ovaries were dissected using high-precision tweezers (IDEAL-TEK, Balerna, Switzerland) in 1X PBS under the Olympus SZX16 stereomicroscope. Dissected ovaries were washed three times with 1X PBS and photographed as described by Zhao et al. (2016). Eggs laid by each pair of *A. hygrophila* adults in the control and treatment groups were collected and counted once. A total of 1,000 eggs were used to calculate the egg hatch rate, and the eggs were assessed every 12 h until they started to decay (Zhao et al., 2016).

### Statistical Analysis

Statistical analyses were performed using SPSS 18.0 (SPSS, Inc., Chicago, IL, United States). All the experimental data are shown as means ± SD (standard deviation). Experimental data were checked for normality and homoscedasticity, and if needed, were arcsine square-root or log-transformed before analysis. Egg hatching rates and ovariole lengths were arcsine-transformed. The number of eggs was square root-transformed. We performed the least significant difference (LSD) test after one-way analysis of variance (ANOVA) to analyze differences in *AhVgR* expression levels between different tissues and developmental stages. *AhVgR* expression levels after injection, ovariole lengths, fecundity of females, and egg hatch rate were analyzed by Student’s *t*-test. We considered means significantly different at *P* < 0.05.

## Results

### Sequence Analysis and Phylogenetic Analysis of the *AhVgR* Gene

**FIGURE 1:**
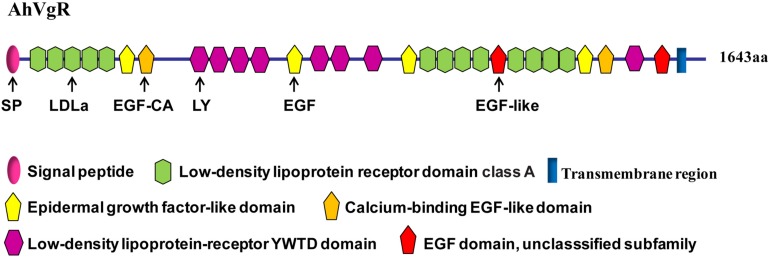
Domain architecture of the *Agasicles hygrophila* vitellogenin receptor (VgR) protein.

The full-length *AhVgR* cDNA was found to be 5,386 bp in length, including a 224 bp 5′ untranslated region (UTR), a 4,929 bp ORF encoding a predicted protein of 1,642 amino acids, and a 233 bp 3′ UTR (GenBank Accession No. MH428915). The theoretical molecular weight is 183.32 kDa, and the pI is 5.08. Analysis of the AhVgR amino acid sequence showed that a 16-amino acid signal peptide (MLVLLLLGVVTPSLGF) is located at the N-terminus of AhVgR. Domain architecture analysis showed that AhVgR is a transmembrane protein with several typical domains, including a LDLR domain class A, an epidermal growth factor (EGF)-like domain, a calcium-binding EGF-like domain, a low-density lipoprotein-receptor YWTD domain, and the transmembrane region (Figure 1). The analysis indicated that AhVgR has three LBDs with five class A (LDLa) cysteine-rich repeats in the first domain and eight in the second domain (Figure 1). Each repeat contains six cysteine residues, and each LDLa is followed by an EGF-like domain. The second and the seventh EGF-like domains contain a calcium-binding domain. The second, third, and seventh EGF-like domains are followed by 4, 3, and 1 repeats of the LDLR YWTD motif, respectively (Figure 1).

**FIGURE 2:**
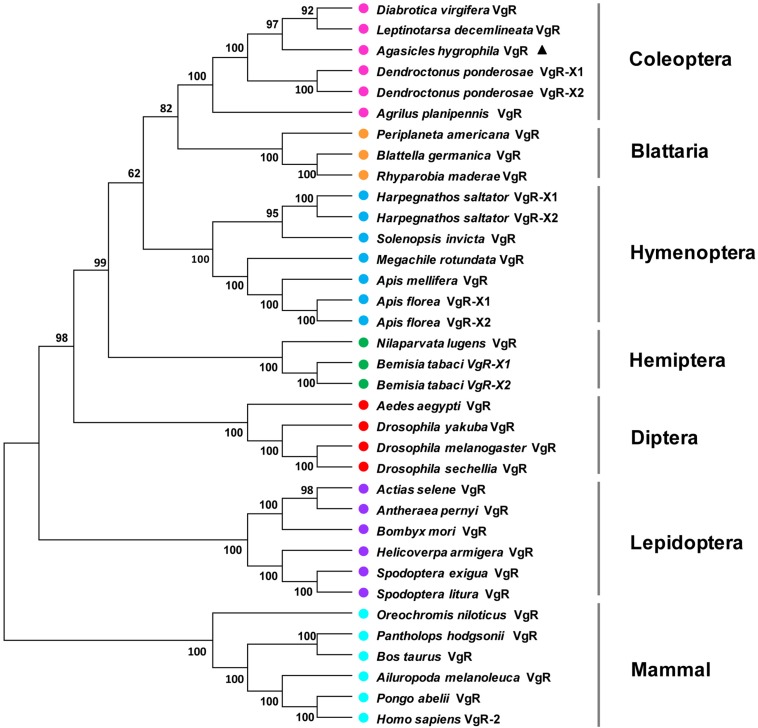
Unrooted consensus neighbor-joining tree showing the evolutionary relationships among vitellogenin receptor proteins. Phylogenetic trees were generated with MEGA 5 using VgR amino acid sequences from insect species in the orders Coleoptera, Hemiptera, Dictyoptera, Lepidoptera, Hymenoptera, and Diptera and five mammal species. The names and accession numbers of the protein sequences used in this analysis are given in Supplementary Table 2.

A phylogenetic analysis of the AhVgR protein showed that VgR sequences from insects are clearly separated from mammalian VgR sequences (Figure 2). In the neighbor-joining tree, VgRs from Coleoptera clustered in one clade, well-separated from VgR proteins from other insect orders, showing that VgRs from Coleoptera all had high amino acid sequence identity and are derived from a common ancestor (Figure 2). The dendrogram also showed that the VgR proteins from *A. hygrophila* and *Leptinotarsa decemlineata* are close, suggesting that the VgRs from these two insects share a close evolutionary relationship. The phylogenetic tree also showed that VgRs from the same insect orders clustered into individual clades, suggesting the conservation of insect VgRs and showing the close evolutionary relationships within the same taxonomic groups. However, the VgRs of Coleoptera clustered most closely with the VgRs of Blattaria with high bootstrap support, showing that the VgRs of these two insect orders share a closer evolutionary relationship than they do with VgRs from other insect orders.

### Tissue-Specific Expression and Temporal Expression Patterns of *AhVgR*

**FIGURE 3:**
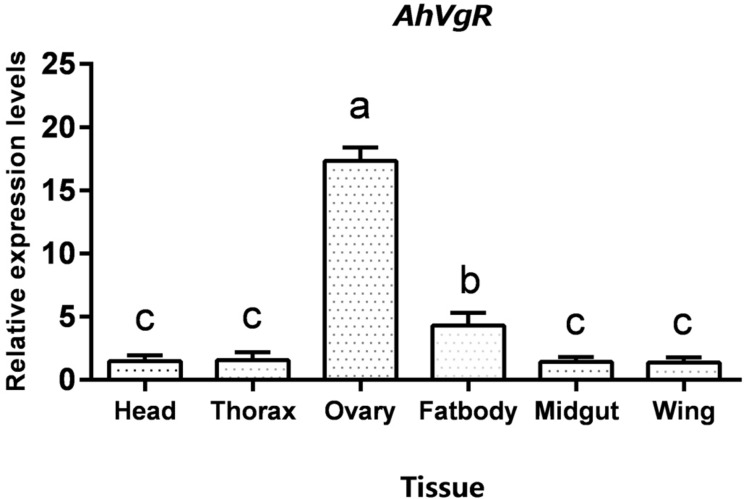
Tissue-specific expression levels of *AhVgR*. The relative mRNA levels were normalized to the expression of the *CoxI* gene and analyzed by the 2^–ΔΔCT^ method. The figure shows data from three replicates that were analyzed by one-way analysis of variance (ANOVA) and the least significant difference (LSD) test. All values are shown as the mean ± SD and bars with different letters indicate significant differences (*P* < 0.05).

**FIGURE 4:**
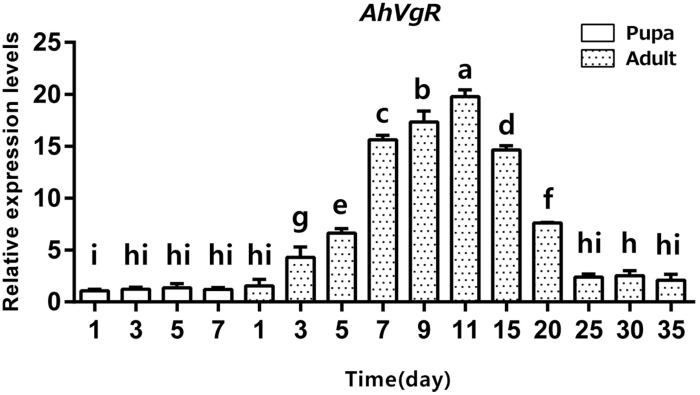
Temporal expression patterns of *AhVgR* in pupae and adults of *A. hygrophila*. The relative mRNA levels were normalized to the expression of the *CoxI* gene and analyzed by the 2^–ΔΔCT^ method. The figure shows data from three replicates that were analyzed by a one-way analysis of variance (ANOVA) and the LSD test. All values are shown as the mean ± SD and bars with different letters indicate significant differences (*P* < 0.05).

qPCR analysis shows that *AhVgR* mRNA is most highly expressed in the ovaries (Figure 3). Analysis of *AhVgR* mRNA expression in the pupal and adult stages revealed that *AhVgR* expression was not detectable in the pupal stage, but was detected in newly-emerged females, reaching a maximum level in 11-day-old individuals (Figure 4). Subsequently, expression decreased gradually to a lower level that was maintained in the adults.

#### Effects of dsRNA Injection on AhVgR Expression

**FIGURE 5:**
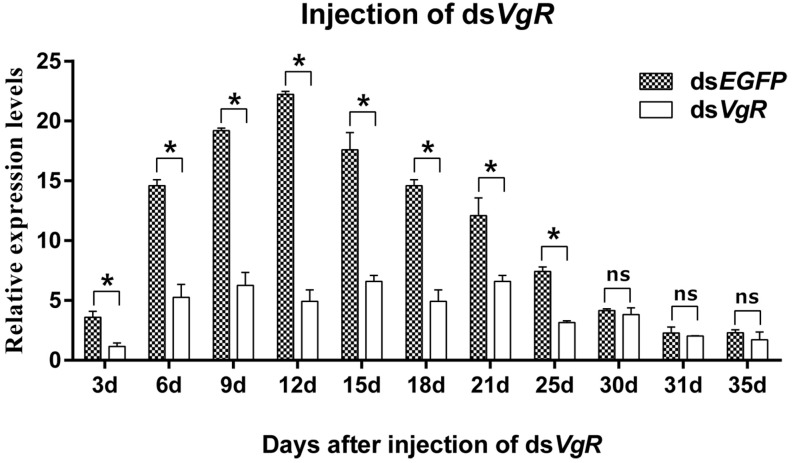
Relative expression of *AhVgR* after injection of ds*VgR* RNA into newly emerged *A. hygrophila* females (*n* = 3). All values are shown as the mean ± SD. The data were analyzed by Student’s *t*-test. ^*^P < 0.05. ns, not significant. ds*EGFP* RNA was used as the control.

qPCR assays showed that *AhVgR* expression in dsVgR-injected females decreased significantly at all sampled time points from 3 to 25 days post-injection (Figure 5). However, *AhVgR* expression did not show an obvious difference between the ds*EGFP*-injected group and ds*VgR*-injected group after 25 days. This may be because of the timeliness of dsRNA. Furthermore, from 3 to 25 days after the injection of ds*AhVgR*, the transcription levels of *AhVgR* decreased by 45.6–81.2% (Figure 5) compared to the ds*EGFP* treatment control group.

#### dsVgR Injection Inhibits Ovarian Development

**FIGURE 6:**
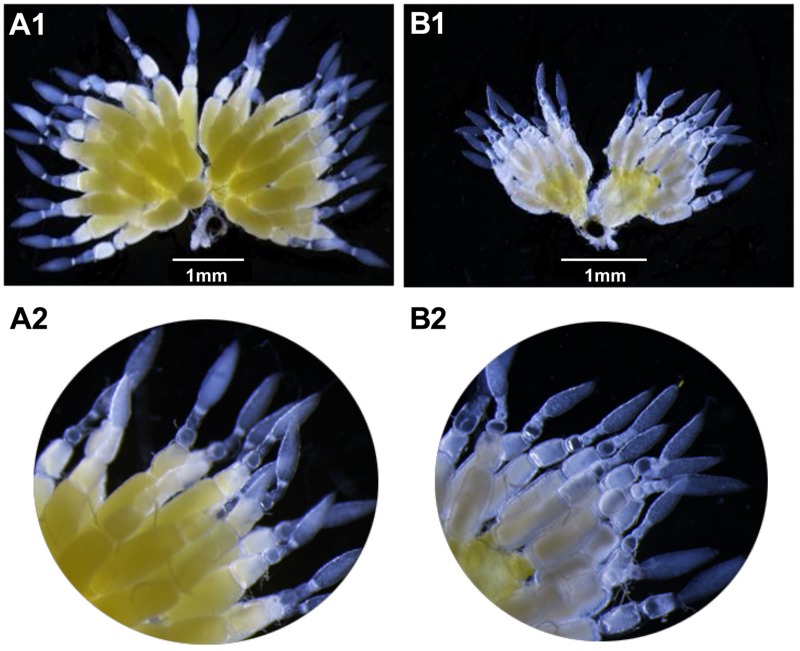
Effect of ds*VgR* on *A. hygrophila* ovary development. **(A1)** It shows an ovary that developed normally. **(A2)** It is an enlargement of **(A1)**. **(B1)** Shows an abnormal ovary that changed morphologically after injection of ds*VgR*. **(B2)** Is an enlargement of **(B1)**.

**FIGURE 7:**
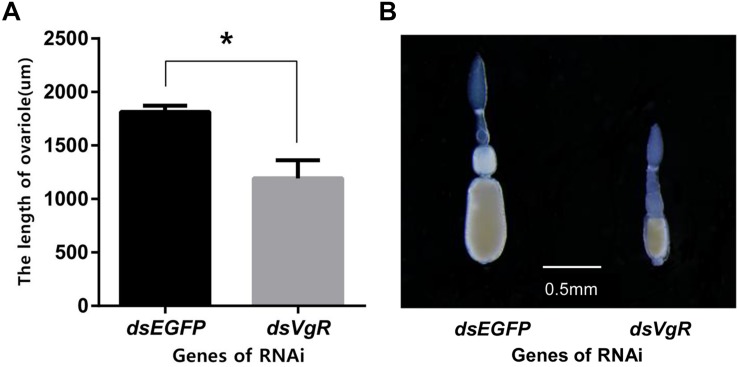
Length of the *A. hygrophila* ovariole after injection with ds*VgR* RNA. **(A)** Ovariole lengths measured from the ds*VgR*-injected and the ds*EGFP*-injected group were analyzed by Student’s *t*-test. ^*^P < 0.05. All values are shown as the mean ± SD. **(B)** Representative ovarioles dissected from the ovaries from the ds*VgR*-injected and ds*EGFP*-injected groups of *A*. *hygrophila* females.

After 4 days, the ovaries of the ds*VgR*-injected group showed a decrease in yolk protein deposition compared to the ds*EGFP*-injected group (Figure 6). The length of the ovariole in the ds*VgR*-injected group was shorter than in the ds*EGFP*-injected group (Figure 7), showing that ovary development in *A. hygrophila* females was inhibited after injection with ds*VgR* RNA, and that AhVgR plays an important role in ovary development and yolk protein deposition.

#### Knockdown of AhVgR Affects Fecundity in *A. hygrophila* Females

**FIGURE 8:**
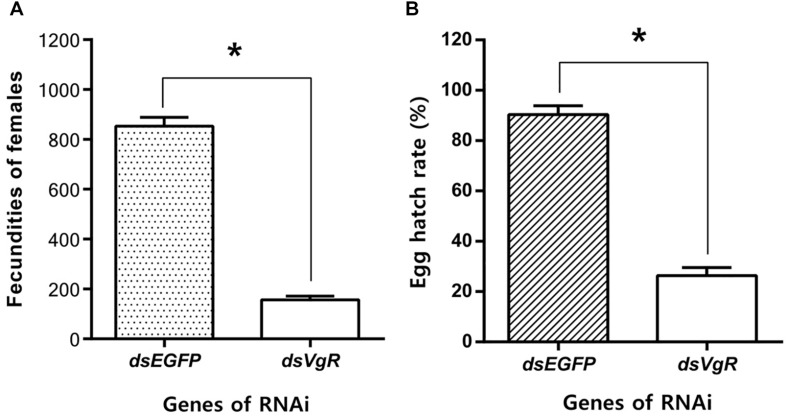
Knockdown of *AhVgR* gene expression reduces fecundity in *A. hygrophila* females. **(A)** Fecundity of females from the ds*VgR*-injected and ds*EGFP*-injected groups (*n* = 20). **(B)** Egg hatch rate from the ds*VgR*-injected and ds*EGFP*-injected groups (*n* = 10). All values are shown as the mean ± SD. The data were analyzed by Student’s *t*-test. ^*^P < 0.05.

After the injection of ds*VgR* RNA into newly-emerged adult females, they laid significantly fewer eggs than did the ds*EGFP*-injected group (Figure 8A). Furthermore, when the hatch rates were compared, eggs produced by the ds*VgR*-injected females had lower hatching success than did eggs from the ds*EGFP*-injected group (Figure 8B). Results of fecundity and egg hatching rate both confirmed that RNA interference of *AhVgR* gene expression significantly inhibited reproduction in *A. hygrophila*.

## Discussion

The *VgR* gene have been studied in many insects, however, this gene has not been characterized in *A. hygrophila*, and studying it is relevant because that this species is used as a biological control agent for the invasive weed *A. philoxeroides*. In our experiments, we characterized *AhVgR* as the LDLR homolog, and analyzed the expression patterns of this gene in different tissues and developmental stages. Our study is the first to use the RNA interference method to explore the function of *AhVgR*, and the results showed that silencing the *AhVgR* gene inhibits ovary development and significantly reduces fecundity in *A. hygrophila.*

VgR proteins belong to the LDLR family, and regulate the process through which Vg enters the oocyte by endocytosis. All oviparous vertebrates and invertebrates have VgR family member proteins (Tufail and Takeda, 2009; Shu et al., 2011). In our study, the full-length cDNA of the *AhVgR* gene was cloned, and the molecular characteristics of this gene were analyzed. The AhVgR protein shares several typical domains with other insect VgRs, such as a LDLR domain class A, an EGF-like domain, a calcium-binding EGF-like domain, and a low-density lipoprotein-receptor YWTD domain. As the structural arrangement found in most insect VgRs, the AhVgR protein contains five cysteine-rich repeats in the first binding site and eight in the second binding site (Figure 1). However, the structural characteristics in other insect VgRs are different. For example, in *Spodoptera litura*, SlVgR has four cysteine-rich repeats in the first binding site and seven in the second binding site. Phylogenetic analysis shows that these insect VgRs cluster into six clades (Coleoptera, Blattaria, Hymenoptera, Hemiptera, Diptera, and Lepidoptera). As expected, AhVgR is in the Coleoptera clade, and the phylogenetic tree indicates that AhVgR shares homology with other Coleoptera VgR proteins, such as those from *Leptinotarsa decemlineata*, *Diabrotica virgifera*, *Dendroctonus ponderosae*, and *Agrilus planipennis* (Figure 2).

In *A. hygrophila*, expression profiles of *AhVgR* showed that this gene is expressed at high levels in the ovaries (Figure 3), indicating that it is an ovary-specific gene, consistent with its role in insect reproduction (Ciudad et al., 2010; Lin et al., 2013). However, *VgR* gene expression has also been detected in other tissues. In *Apis mellifera*, *AmVgR* expression was detected in the hypopharyngeal gland and other tissues (Guidugli-Lazzarini et al., 2008). The various expression patterns of *VgR* genes in different tissues may be related to their ligand Vg, which regulates longevity and the juvenile hormone titer in bees (Corona et al., 2007; Nelson et al., 2013). The temporal expression patterns of *AhVgR* were found to be closely correlated with the reproductive process, and expression levels significantly increased in the ovaries beginning 3 days after emergence (Figure 4). This pattern is consistent with *VgR* expression patterns in other insects, such as in *Nilaparvata lugens*, *Thitarodes pui*, and *Aphis citricidus* (Lu et al., 2015; Shang et al., 2018; Wu et al., 2018).

RNA interference is considered to be a specific tool to efficiently determine gene functions (Hannon, 2002) and also to control gene expression at the mRNA level (Fire et al., 1998). We used RNAi to explore the functions of AhVgR *in vivo*. The results showed that injection with ds*VgR* RNA displayed high efficiency and caused significant differences in fecundity and ovarian development compared to the control group (Figures 6–8). *AhVgR* expression decreased after the ds*VgR* treatment, and the effects of ds*VgR* RNA lasted for about 25 days after injection (Figure 5). The high gene silencing efficiency of dsRNA has also been verified in other Coleoptera species such as *Tribolium castaneum*, *Leptinotarsa decemlineata*, and *Diabrotica virgifera* (Arakane et al., 2005; Shi et al., 2016; Camargo et al., 2018). After injecting the ds*VgR* RNA, the ovaries exhibited a decrease in yolk protein deposition compared to the control group (Figure 6), and the lengths of the ovarioles were also reduced (Figure 7). Furthermore, we observed that *A. hygrophila* fecundity, as measured by the number of eggs laid and their hatch rate, both decreased sharply after injection with ds*VgR* RNA (Figure 8). Our results are consistent with an earlier study which suggested that the capability of reproduction in insects depends on two primary steps, (1) the process of vitellogenin (Vg) formation and deposition and (2) transport of the Vg to oocytes by the vitellogenin receptor (VgR) (Sappington and Raikhel, 1998). The functions of VgR have been also explored in other insect species in which the functions of Vg have been studied, and the results are in accordance with ours (Shu et al., 2011; Lin et al., 2013; Shang et al., 2018).

## Conclusion

In our study, the molecular characterization of *AhVgR* and the expression patterns of this gene in different tissues and developmental stages were analyzed. Analysis of amino acid sequences of AhVgR suggests AhVgR is a member of the LDLR family and displays high sequence similarity to VgRs from other insects. The tissue and developmental stage-specific mRNA expression patterns of *AhVgR* were also similar to those in other insects. We found that *AhVgR* was most highly expressed in the ovary of adult stage. After the basic characterization of the gene, we explored the functions of AhVgR via RNA interference, and the results indicate that the AhVgR protein is necessary to stimulate yolk uptake, as well as being critical for ovary development and egg laying in *A. hygrophila*.

## Data Availability

All datasets generated for this study are included in the manuscript and/or the Supplementary Files.

## Author Contributions

HZ and JG conceived and designed the experiments, and wrote the manuscript. HZ performed the experiments. HZ, YL, JJ, and ZZ analyzed the data.

## Conflict of Interest Statement

The authors declare that the research was conducted in the absence of any commercial or financial relationships that could be construed as a potential conflict of interest.

## Footnotes

1 http://www.ncbi.nlm.nih.gov/BLAST/

2 https://www.ncbi.nlm.nih.gov/orffinder/

3 http://www.expasy.org

4 http://smart.embl-heidelberg.de/

5 http://www.cbs.dtu.dk/services/SignalP
