# Supplementary material for: Identification and Characterization of the Vitellogenin Receptor Gene and Its Role in Reproduction in the Alligatorweed Flea Beetle, Agasicles hygrophila
Source: Front Physiol. 2019 Jul 31;10:969. doi: 10.3389/fphys.2019.00969 (PMC6684774; doi:10.3389/fphys.2019.00969)
Supplement: Supplementary file 1 [file Table_1.DOC]

Supplementary Information for

**Identification and Characterization of the Vitellogenin Receptor Gene and its Role in Reproduction in the alligatorweed flea beetle, *Agasicles hygrophila***

**Hong Zhang, Yiran Liu, Jisu Jin, Zhongshi Zhou, Jianying Guo***

State Key Laboratory for Biology of Plant Diseases and Insect Pests, Institute of Plant Protection, Chinese Academy of Agricultural Sciences, Beijing, China

* Corresponding author: Jianying Guo, State Key Laboratory for Biology of Plant Diseases and Insect Pests, Institute of Plant Protection, Chinese Academy of Agricultural Sciences, # 2 West Road of Yuanmingyuan, Haidian, Beijing 100193, China. Tel/Fax: +86 10 62815910; E-mail: [guojianying@caas.cn](mailto:guojianying@caas.cn)

Supplementary Tables

Supplementary Table 1. List of oligonucleotide primers used for cDNA cloning, real-time quantitative PCR assays, and dsRNA synthesis.

Supplementary Table 2. Protein names, species names, and sequences of the VgR proteins used in Figure 2.

Supplementary Table 1. List of primers used for cDNA cloning, real-time quantitative PCR, and dsRNA synthesis.

| Primer name | Primer sequence (5’-3’) | Length of PCR product |
| --- | --- | --- |
| *AhVgR*-F | ACTGGACAGTATACCTGCAGTAA | 4749 bp |
| *AhVgR*-R | TCCTGGCTGTAGGAATGG |
| *AhVgR-*5’RACE | GATATGCATTTGTTGTTCTTACATCTG | 466 bp |
| *AhVgR-*3’RACE | GGACCAATTGCTGCTGCGATTGTAC | 500 bp |
| qPCR*-AhCoxI-*F | TTAACGGGAGTGGTCTTAG | 107 bp |
| qPCR*-AhCoxI-*R | GCAAATACAGCTCCTATAGATAG |
| qPCR*-AhVgR-*F | CGTTGAAGAAGGTGATATGTATTG | 124 bp |
| qPCR*-AhVgR-*R | TAGACCATTTGGCCAATGTATG |
| ds*EGFP-*A-F | TAATACGACTCACTATAGGGCATGAAGCAGCACGACTT | 433 bp |
| ds*EGFP-*A-R | TAATACGACTCACTATAGGGCAGCAGGACCATGTGATC |
| ds*EGFP-*B-F | TAATACGACTCACTATAGGGGAGGCCCGCACCGATCG | 412 bp |
| ds*EGFP-*B-R | TAATACGACTCACTATAGGGCCTCGGCGCGGGTCTTGTAG |
| ds*VgR-*A-F | TAATACGACTCACTATAGGGAGCCAACGATCTTGTTTAG | 562 bp |
| ds*VgR-*A-R | TAATACGACTCACTATAGGGGATCATGATCGTAAGTCAATG |
| ds*VgR-*B-F | TAATACGACTCACTATAGGGATGCAAGTATCCCAAGC | 502 bp |
| ds*VgR-*B-R | TAATACGACTCACTATAGGGTCCTTTGGCTTTGCAGGAT |

Supplementary Table 2. Protein names and sequences of the VgR sequences used in Figure 2.

| Protein name | Accession number of protein sequences |
| --- | --- |
| *Bemisia tabaci* VgR*-X1* | XP_018899796.1 |
| *Bemisia tabaci* VgR*-X2* | XP_018899797.1 |
| *Nilaparvata lugens* VgR | ADE34166.1 |
| *Blattella germanica* VgR | CAJ19121.1 |
| *Periplaneta americana* VgR | BAC02725.2 |
| *Rhyparobia maderae* VgR | BAE93218.1 |
| *Actias selene* VgR | AFV32171.1 |
| *Antheraea pernyi* VgR | AEJ88360.1 |
| *Bombyx mori* VgR | ADK94452.1 |
| *Helicoverpa armigera* VgR | AGF33811.2 |
| *Spodoptera exigua* VgR | AOX13593.1 |
| *Spodoptera litura* VgR | ADK94033.1 |
| *Apis florea* VgR*-X1* | XP_012350206.1 |
| *Apis florea* VgR*-X2* | XP_012350207.1 |
| *Apis mellifera* VgR | XP_016767970.1 |
| *Harpegnathos saltator* VgR*-X1* | XP_011139074.1 |
| *Harpegnathos saltator* VgR*-X2* | XP_025153783.1 |
| *Megachile rotundata* VgR | XP_012143749.1 |
| *Solenopsis invicta* VgR | AAP92450.1 |
| *Agrilus planipennis* VgR | XP_018318647.1 |
| *Dendroctonus ponderosae* VgR*-X1* | XP_019758255.1 |
| *Dendroctonus ponderosae* VgR*-X2* | XP_019758256.1 |
| *Diabrotica virgifera VgR* | AQS83398.1 |
| *Leptinotarsa decemlineata VgR* | XP_023027421.1 |
| *Agasicles hygrophila* VgR | MH428915 |
| *Aedes aegypti* VgR | AAC28497.1 |
| *Drosophila melanogaster* VgR | AAB60217.1 |
| *Drosophila sechellia* VgR | XP_002042769.1 |
| *Drosophila yakuba* VgR | XP_002100545.2 |
| *Ailuropoda melanoleuca* VgR | XP_002916562.1 |
| *Pantholops hodgsonii* VgR | XP_005977011.1 |
| *Bos taurus* VgR | DAA32788.1 |
| *Oreochromis niloticus* VgR | XP_005453016.1 |
| *Pongo abelii* VgR | XP_002812613.1 |
| *Homo sapiens* VgR-2 | EAX11280.1 |
